# Electromagnetic Force on an Aluminum Honeycomb Sandwich Panel Moving in a Magnetic Field

**DOI:** 10.3390/s23208577

**Published:** 2023-10-19

**Authors:** Yunfeng Yu, Honghao Yue, Feiyang Wen, Haihong Zhao, Aiyu Zhou

**Affiliations:** 1 Aerospace System Engineering Shanghai, Shanghai 201109, China; yf_yu21@163.com (Y.Y.); 15104598145@163.com (A.Z.); 2 State Key Laboratory of Robotics and System, Harbin Institute of Technology, Harbin 150001, China; 21B908105@stu.hit.edu.cn (F.W.); zhaohaihong@hit.edu.cn (H.Z.)

**Keywords:** aluminum honeycomb sandwich panel, electromagnetic force, equivalent conductivity tensor, electromagnetic detumbling

## Abstract

This paper reports a method for calculating the electromagnetic force acting on an aluminum honeycomb sandwich panel moving in a magnetic field. This research is motivated by the non-contact electromagnetic detumbling technology for space non-cooperative targets. Past modeling of the electromagnetic forces and torques generally assumes that the target is homogeneous. However, aluminum honeycomb sandwich panels are extensively used in spacecraft structures to reduce weight without sacrificing structural strength and stiffness, which are so inhomogeneous and complicated that it is difficult to obtain the induced electromagnetic force even by numerical methods. An equivalent conductivity tensor of an aluminum honeycomb sandwich panel is proposed, which allows the aluminum honeycomb sandwich panel to be treated as a homogeneous structure when calculating the induced electromagnetic forces. The advantage of the equivalent conductivity tensor in the calculation of induced electromagnetic forces is verified by finite element simulations. The proposed method makes it possible to evaluate the electromagnetic force of a large aluminum honeycomb sandwich structure moving in a magnetic field.

## 1. Introduction

On-orbit service is of great economic benefit by rescuing malfunctioning satellites or removing space debris from some vital orbits. These targets that need to be served or removed are generally non-cooperative and have complex rotations. Partial observations of the angular rate of space debris are shown in Table 1. The huge rotating energy of the target increases the risk of collision between the servicing spacecraft and the target. Servicing spacecraft have to consider how to decrease the rotational speed of the targets if they want to successfully capture and dock with the target.

**Figure 1:**
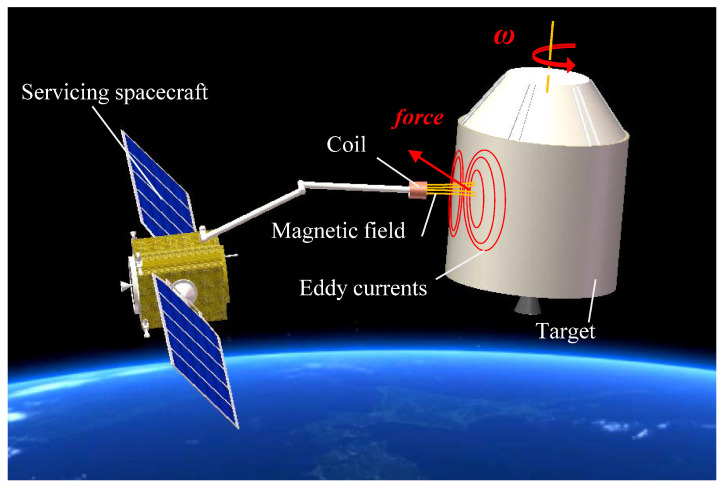
Electromagnetic detumbling conceptual diagram.

Electromagnetic de-tumbling is a potential option due to its non-contact nature and low risk of collision. The servicing spacecraft uses the onboard electromagnetic device to generate a magnetic field near the rotating target, and when the target cuts the magnetic line, its conductor structures will generate currents, also known as eddy currents, which interact with the primary magnetic field to generate an electromagnetic force that hinders the relative motion (see Figure 1). In recent decades, many innovative and potential electromagnetic de-tumbling designs have been proposed [1,2,3]. Study [4] described a de-tumbling strategy of using two robotic arms equipped with eddy current brakes to apply an external magnetic field near the target surface. Li et al. [5] presented a method of using a robotic arm, which is equipped with an electromagnetic coil at the end to break an uncontrolled satellite. One or more high-temperature superconducting coils were proposed to be placed several meters away from the surface of space debris to apply an external magnetic field in Refs. [6,7,8]. Refs. [9,10] discussed the feasibility of using a rotating magnetic field to de-tumbling malfunctioning satellites. However, there are still many challenges in the electromagnetic de-tumbling of non-cooperative targets in orbits.

**Table 1:** Partial observations of the angular rate of space debris.

Spacecraft	Orbit	Rotation Angular Rate (Time)	Reference
GOES 8	GEO	22.4${}^{\circ }$/s (12 December 2013) 21.84${}^{\circ }$/s (27 February 2014) 4.76${}^{\circ }$/s (25 July 2014)	[11]
GOES 10	GEO	15.6${}^{\circ }$/s (28 February 2014) 11.1${}^{\circ }$/s (19 March 2014) 13.7${}^{\circ }$/s (28 August 2014)	[11]
BSAT 1A	GEO	114.87${}^{\circ }$/s (15 April 2014) 115.42${}^{\circ }$/s (28 March 2015) 115.5${}^{\circ }$/s (11 September 2015)	[12]
Brazilsat B1	GEO	94.22${}^{\circ }$/s (28 March 2015)	[12]
KOREASAT 1	GEO	4.9${}^{\circ }$/s (23 June 2013)	[13]
BREEZE-M body	HEO	400.9${}^{\circ }$/s (6 January 2004)	[14]
INTELSAT 4-F7	GEO	139${}^{\circ }$/s (6 January 2004)	[14]
GLONAS satellite	GEO	1.1${}^{\circ }$/s–42.2${}^{\circ }$/s	[14]
ASTRO-H	LEO	69${}^{\circ }$/s (28 March 2016)	[13]
Cosmos 2082 body	LEO	8.8${}^{\circ }$/s (6 January 2004)	[15]
ENVISAT	LEO	1.96${}^{\circ }$/s (28 April 2016)	[14]
ADEOS-2	LEO	4.3${}^{\circ }$/s (30 March 2016)	[14]

One of these challenges is to model and evaluate the induced electromagnetic force and torque acting on the target, which provides the input for eddy current brake design and dynamic analysis of the de-tumbling system. There are only few cases where analytical expressions for electromagnetic forces can be determined. Generally, a finite element method is required. In the existing research, the target is generally regarded as a homogeneous aluminum geometry, such as a homogeneous sphere, a homogeneous cylinder, or a homogeneous cube. Smith et al. [16,17] gave an analytical method for calculating the induced electromagnetic torque on a homogeneous spherical shell and cylindrical shell rotating in a uniform magnetic field. Youngquist [18,19] discusses the electromagnetic force and torque on a homogeneous sphere rotating in an axisymmetric magnetic field. We presented an approximate calculation method of the induced electromagnetic force and torque acting on a rotating homogeneous conducting sphere, cube, and cylinder in a single or double magnetic dipole magnetic field [7]. Some researchers have explored numerical methods for solving the electromagnetic force and torque on homogeneous structures [5,6,20].

In fact, a large number of aluminum honeycomb sandwich panels are extensively used in spacecraft structures due to their high strength, stiffness, and low mass. The electromagnetic force and torque acting on a moving aluminum honeycomb sandwich panel in a magnetic field are rarely studied. Building and solving finite element models of these structures remain challenging due to the complexity of the geometries. The complex honeycomb core structure was directly ignored, and only the homogeneous skins are considered in ref. [21], which obviously has limitations and is not applicable to all types of aluminum honeycomb sandwich panels.

The present paper introduces a method for calculating the induced electromagnetic force acting on a moving aluminum honeycomb sandwich panel in a magnetic field. Section 2 introduces the basic principles of electromagnetic detumbling, and Section 3 introduces an equivalent conductivity tensor of the honeycomb sandwich panel. In Section 4, the finite element models and numerical calculation results are presented. In Section 5, the feasibility of the calculation method for the electromagnetic force is verified by experiments. Section 6 gives the conclusions.

## 2. Basic Principles of Electromagnetic Detumbling

When a conductor moves in a magnetic field, eddy currents will be induced in it, which can be described as

(1) j=σ[−∇ϕ+v×B]

where σ is the conductivity tensor of the conductor, ϕ is the electric potential, v is the velocity of the conductor relative to the primary magnetic field, and B is the primary magnetic field.

The charge is a conserved quantity and it complies with the continuity equation

(2) $$\nabla \cdot j=-\frac{\partial \rho }{\partial t}$$

where ρ is the charge density.

The characteristic time of the transient state of the charge building on the conductive object before the currents form closed loops can be neglected for conductive materials. The induction equation simplifies to the so-called quasi-static approximation, and the continuity Equation (2) reduces to

(3) ∇·j=0

Combining Equations (1) and (3), the electric potential should satisfy Poisson’s Equation (4), which has to be solved to determine j.

(4) $$\nabla^{2}\phi =\nabla \cdot \left(v\times B\right)$$

If the conductor is rotating around a fixed point, Equation (4) can be converted to

(5) $$\nabla^{2}\phi =2\omega \cdot B$$

The eddy currents only exist inside the conductor, the normal components of eddy currents on the conductor surface are zero, and the induced potential should satisfy the boundary conditions.

(6) $$\frac{\partial \phi }{\partial n_{\Gamma }}{|}_{\Gamma }=\left(v\times B\right)\cdot n_{\Gamma }=0$$

where $n_{\Gamma }$ denotes the surface normal vector of the surface Γ.

The Lorentz force density acting on the conductor is given by

(7) f=j×B

The electromagnetic force acting on the conductor is given by the volume integral Equation (8):

(8) $$F=\int_{V}fdV=\int_{V}j\times BdV$$

The electromagnetic torque acting on the conductor is given by the volume integral Equation (9):

(9) $$T=\int_{V}r\times fdV=\int_{V}r\times \left(j\times B\right)dV$$

where r denotes the position vector of the field point relative to the centroid of the conductor.

In fact, there are only a few cases where the solution can be analytically determined. Most of the time, a finite element method or a finite difference method must be used [20].

Aluminum honeycomb sandwich panels have many fine and complex structures, resulting in difficult meshing and huge computational effort for solving. In the following, An equivalent conductivity tensor is introduced to make the calculation of induced electromagnetic force easy for large aluminum honeycomb panels.

## 3. Equivalent Conductivity Tensor

**Figure 2:**
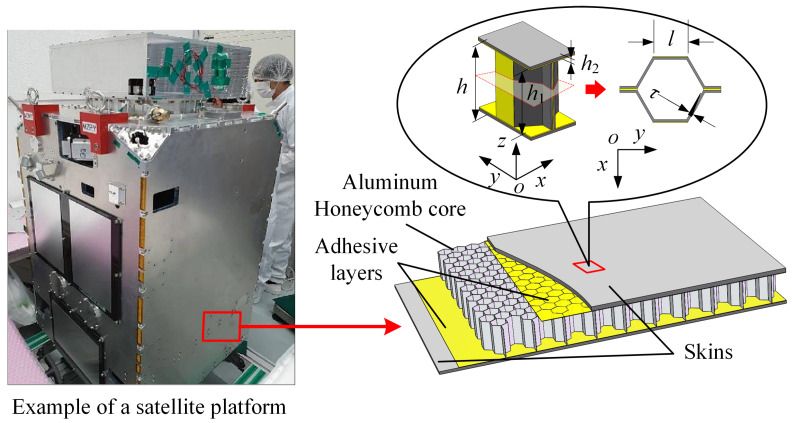
Electromagnetic detumbling conceptual diagram.

An aluminum honeycomb sandwich panel is shown in Figure 2, which is mainly composed of an aluminum honeycomb core, adhesive layers, upper and lower skins. The skins of aluminum honeycomb sandwich panels used in spacecraft are generally fabricated from aluminum alloy or carbon fiber-reinforced plastic (CFRP).

**Figure 3:**
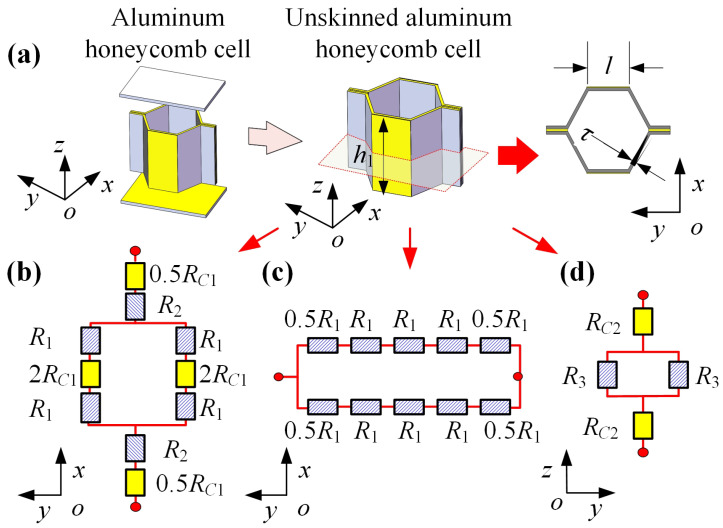
Equivalent circuits of an unskinned aluminum honeycomb cell. (**a**) Aluminum honeycomb cell, (**b**) equivalent circuit of an aluminum honeycomb cell in x-axis direction, (**c**) equivalent circuit of an aluminum honeycomb cell in y-axis direction, (**d**) equivalent circuit of an aluminum honeycomb cell in z-axis direction.

Taking a hexagonal honeycomb sandwich panel as an example, it can be divided into a number of aluminum honeycomb cells. The equivalent circuits of an unskinned aluminum honeycomb cell in three directions are shown in Figure 3.

The equivalent resistances in the three directions can be expressed as

(10a) $$R_{x}=R_{1}+2R_{2}+2R_{C1}$$

(10b) $$R_{y}=2R_{1}$$

(10c) $$R_{z}=0.5R_{3}+2R_{C2}$$

where $R_{C1}$ is the resistance of the adhesive layer between the foils; $R_{C2}$ is the resistance of the adhesive layer between the foil and the skin; and $R_{1}$, $R_{2}$, and $R_{3}$ are given by Equation (11):

(11a) $$R_{1}=\frac{1}{\sigma_{1}}\frac{l}{h_{1}\tau }$$

(11b) $$R_{2}=\frac{1}{\sigma_{1}}\frac{\tau }{h_{1}l}$$

(11c) $$R_{3}=\frac{1}{\sigma_{1}}\frac{h_{1}}{4l\tau }$$

where $\sigma_{1}$ is the conductivity of aluminum foils, *h* is the foil height, τ is the foil thickness, and *l* is the cell length.

Approximately treating the honeycomb core as a homogeneous geometry, its equivalent conductivity tensor can be expressed as

(12) $${\bar{\sigma }}_{\mathrm{core}}=\left[\begin{array}{ccc} \frac{1}{R_{x}}\frac{\sqrt{3}l}{3lh_{1}} & 0 & 0\\ 0 & \frac{1}{R_{y}}\frac{3l}{\sqrt{3}lh_{1}} & 0\\ 0 & 0 & \frac{1}{R_{z}}\frac{h_{1}}{\sqrt{3}l\left(3l\right)} \end{array}\right]$$

Substituting Equations (10) and (11) into Equation (12) yields

(13) $${\bar{\sigma }}_{\mathrm{core}}\;=\;\left[\;\begin{array}{ccc} \frac{\sqrt{3}l\tau \sigma_{1}}{3l^{2}\;+\;6\tau^{2}\;+\;6h_{1}\tau lR_{C1}\sigma_{1}}\; & \;0\; & \;0\;\\ \;0\; & \;\frac{\sqrt{3}\tau \sigma_{1}}{2l}\; & \;0\;\\ \;0\; & \;0\; & \frac{8\sqrt{3}h_{1}\tau \sigma_{1}}{9l\;\left(h_{1}\;+\;16l\tau R_{C2}\sigma_{1}\right)} \end{array}\right]$$

The conductivity tensor of an aluminum skin or a CFRP skin can be expressed as [22,23]

(14) $$\begin{array}{c} \sigma_{\mathrm{skin}}=\left[\begin{array}{ccc} \sigma_{11}^{} & 0 & 0\\ 0 & \sigma_{22}^{} & 0\\ 0 & 0 & \sigma_{33}^{} \end{array}\right] \end{array}$$

For aluminum skin, the elements on the diagonal of the conductivity tensor $\sigma_{\mathrm{skin}}$ are equal, i.e.,

(15) $$\sigma_{11}=\sigma_{22}=\sigma_{33}$$

According to the mixing rule, the equivalent conductivity tensor of an aluminum honeycomb sandwich panel can be expressed as

(16) $$\bar{\sigma }={\bar{\sigma }}_{\mathrm{core}}V_{1}+\sigma_{\mathrm{skin}}V_{2}$$

where $V_{1}$, $V_{2}$ are, respectively, the volume fractions of the equivalent geometry of the aluminum honeycomb core and skins, which can be determined by Equations (17) and (18).

(17) $$\begin{array}{c} V_{1}=\frac{h_{1}}{h}=\frac{h_{1}}{h_{1}+2h_{2}} \end{array}$$

(18) $$\begin{array}{c} V_{2}=\frac{2h_{2}}{h}=\frac{2h_{2}}{h_{1}+2h_{2}} \end{array}$$

Substituting Equations (13), (14), (17) and (18) into Equation (16), the expression of the equivalent conductivity tensor of the aluminum honeycomb sandwich panel can be obtained as

(19) $$\bar{\sigma }\;=\;\frac{1}{h}\;\left[\begin{array}{ccc} \frac{l\tau h_{1}\sigma_{1}}{\sqrt{3}\left(l^{2}\;+\;2\tau^{2}\;+\;2h_{1}l\tau R_{C1}\sigma_{1}\right)}\;+\;2h_{2}\sigma_{11} & 0 & 0\\ 0 & \frac{\sqrt{3}\tau h_{1}\sigma_{1}}{2l}\;+\;2h_{2}\sigma_{22} & 0\\ 0 & 0 & \frac{8\sqrt{3}h_{1}^{2}\tau \sigma_{1}}{9l\left(h_{1}\;+\;16l\tau R_{C2}\sigma_{1}\right)}\;+\;2h_{2}\sigma_{33} \end{array}\right]$$

It can be seen that the resistances of the adhesive layers can affect the conductivity in the *x*-axis and *z*-axis directions; however, they will not affect the conductivity in the *y*-axis direction because there are no adhesive layers in the *y*-axis direction.

Considering that the adhesive layers of aluminum honeycomb sandwich panels for aerospace are generally very thin and that some metal-embedded blocks used for connection are often embedded in the aluminum honeycomb sandwich panels, the influence of the adhesive layers on the conductivity is very small. Ignoring the resistances of the adhesive layers and considering $\frac{\tau }{l}\ll 1$ for the aluminum foils, Equation (19) can be simplified as

(20) $$\bar{\sigma }\;=\;\left[\begin{array}{ccc} \frac{\sqrt{3}\tau \sigma_{1}\left(h-2h_{2}\right)}{3lh}\;+\;\frac{2h_{2}}{h}\sigma_{11} & 0 & 0\\ 0 & \frac{\sqrt{3}\tau \sigma_{1}\left(h-2h_{2}\right)}{2lh}\;+\;\frac{2h_{2}}{h}\sigma_{22} & 0\\ 0 & 0 & \frac{8\sqrt{3}\tau \sigma_{1}\left(h-2h_{2}\right)}{9lh}\;+\;\frac{2h_{2}}{h}\sigma_{33} \end{array}\right]$$

## 4. Finite Element Models and Numerical Calculation Results

**Figure 4:**
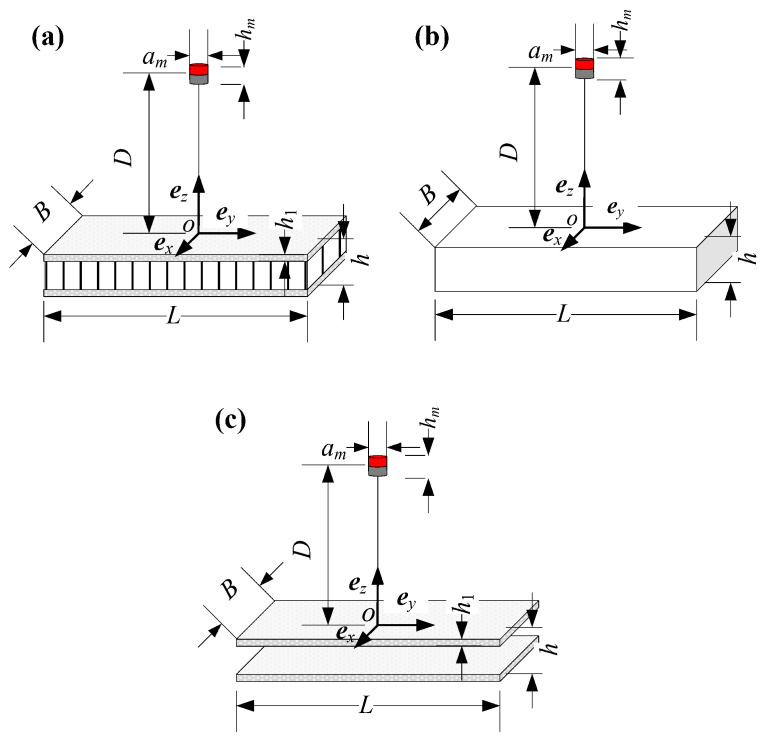
The three geometries considered in finite element models. (**a**) An intact aluminum honeycomb sandwich panel moving relative to a cylindrical permanent magnet. (**b**) A homogeneous panel moving relative to a cylindrical permanent magnet. (**c**) Two skins moving to a cylindrical permanent magnet.

Since the expression of the equivalent conductivity tensor of an aluminum honeycomb sandwich panel has been derived, the aluminum honeycomb sandwich panel has the potential to be equivalent to a homogeneous geometry to solve for electromagnetic forces. Finite element models of the three geometries shown in Figure 4 are established to obtain the induced electromagnetic forces. Figure 4a shows that an intact aluminum honeycomb sandwich panel moves relative to a cylindrical permanent magnet, Figure 4b shows that a homogeneous panel with the same size as the aluminum honeycomb sandwich panel moves relative to a cylindrical permanent magnet, and Figure 4c shows that two skins without aluminum honeycomb cores move relative to a cylindrical permanent magnet. These three geometries move with a velocity *v* in the negative *x*-axis direction.

**Figure 5:**
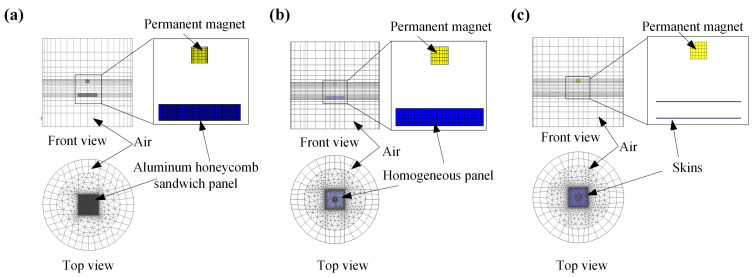
Finite element discretization of the geometries. (**a**) The finite element discretization of the aluminum honeycomb sandwich panel. (**b**) The finite element discretization of the homogeneous panel. (**c**) The finite element discretization of the two skins.

The values of parameters used in the finite element models are shown in Table 2. The conductivity tensor of the homogeneous panel is determined by Equation (20) based on the geometry and material of the aluminum honeycomb sandwich panel. The grids of the three finite element models are shown in Figure 5.

The outer boundary of the air domains is magnetic insulation. The adhesive layers of the aluminum honeycomb sandwich panel are ignored to reduce the difficulty of modeling and mesh generation. Even so, more than 1.31 million volume grids are generated in the finite element model of the intact honeycomb sandwich panel due to the existence of the thin foils, which are about 21 times that of the homogeneous panel and 22 times that of the thin skins. Therefore, it is more expensive to solve the finite element model of the intact aluminum honeycomb sandwich panel moving a magnet field, which limits the numerical solution of the induced electromagnetic force acting on some larger aluminum honeycomb sandwich panels directly.

### 4.1. Case One: Aluminum Skins

Assume that the conductivity of the aluminum foils is $\sigma_{1}=3.5\times 10^{7}$ S/m. The skins are assumed to be fabricated from aluminum and the conductivity of them is the same as that of the aluminum foils; thus, Equation (21) can be obtained.

(21) $$\sigma_{11}=\sigma_{22}=\sigma_{33}=3.5\times 10^{7}\;S/m$$

According to Equation (20), the equivalent conductivity tensor of the aluminum honeycomb sandwich panel with aluminum skins can be obtained as

(22) $$\bar{\sigma }=\left[\begin{array}{ccc} 2.4799 & 0 & 0\\ 0 & 2.6698 & 0\\ 0 & 0 & 3.1131 \end{array}\right]\times 10^{6}\;S/m$$

**Figure 6:**
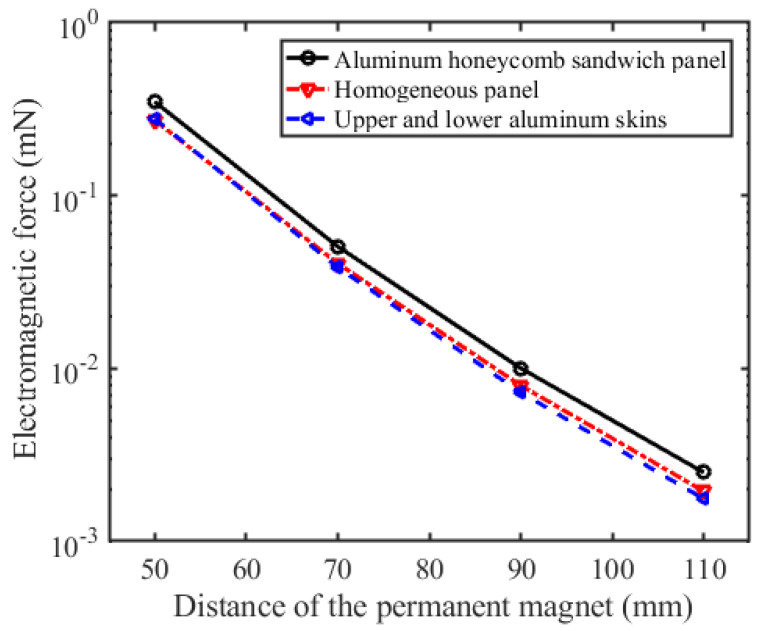
Numerical results of the induced electromagnetic forces.

The numerical results of the induced electromagnetic force on the three geometries are shown in Figure 6.

It can be seen that when the skins of the aluminum honeycomb sandwich panels are fabricated from aluminum, the numerical results of the induced electromagnetic forces of the three geometries have little difference. In this case, the skins can replace the aluminum honeycomb sandwich panel to calculate the induced electromagnetic force, and the contribution of the aluminum honeycomb core to the induced electromagnetic force can be ignored, which is consistent with the experimental conclusion in ref. [21].

### 4.2. Case Two: CFRP Skins

Assume that the conductivity of the aluminum foils is also $\sigma_{1}=3.5\times 10^{7}$ S/m. The conductivity of the CFRP skin is related to the type, ratio, arrangement direction, and forming process of carbon fiber, and there is a certain anisotropy. Generally, the many multilayer CFRPs arranged orthogonally have similar conductivity in plane, and the conductivity is in the order of $10^{6}$ S/m. Ignoring the anisotropic differences in the conductivity of the CFRP skins, the conductivity of the CFRP skins here is assumed to be

(23) $$\sigma_{11}=\sigma_{22}=\sigma_{33}=2.5\times 10^{6}\;S/m$$

According to Equation (20), the equivalent conductivity tensor of the aluminum honeycomb sandwich panel with CFRP skins is given by

(24) $$\bar{\sigma }=\left[\begin{array}{ccc} 0.5299 & 0 & 0\\ 0 & 0.7198 & 0\\ 0 & 0 & 1.1631 \end{array}\right]\times 10^{6}\;S/m$$

**Figure 7:**
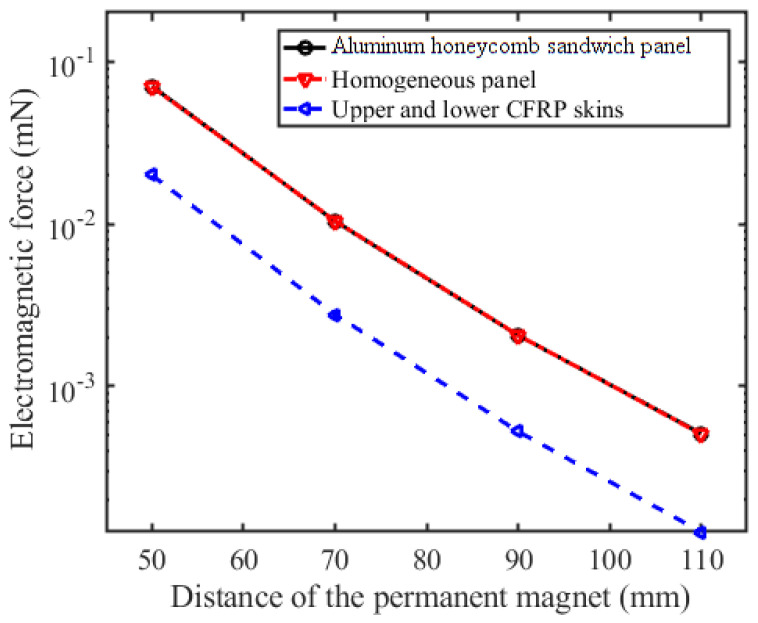
Numerical results of the induced electromagnetic force.

Figure 7 shows the numerical results of the induced electromagnetic force on the three geometries when the skins of the aluminum honeycomb sandwich panel are fabricated of CFRP. The relative error of the induced electromagnetic forces on the skins without an aluminum honeycomb core is more than 70% compared with that of the intact aluminum honeycomb sandwich panel. Nevertheless, the result of the induced electromagnetic force of the homogeneous panel is not more than 1%.

Therefore, the contribution of the aluminum honeycomb core to the induced electromagnetic force cannot be ignored when the aluminum honeycomb sandwich panel is skinned with CFRP skins. The equivalent homogeneous geometry can obtain better accuracy.

## 5. Experiment

### 5.1. Experiment Up

**Figure 8:**
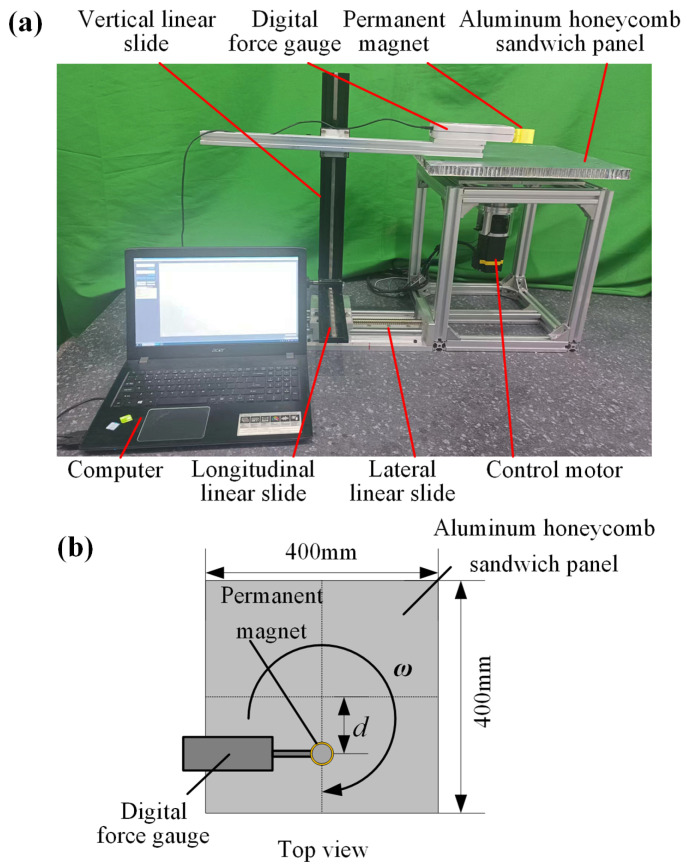
Experimental setup for the induced electromagnetic force measurement of the aluminum honeycomb sandwich panel. (**a**) Photo of the experimental setup. (**b**) Schematic diagram of the position of the permanent magnet relative to the tested aluminum honeycomb sandwich panel.

To verify the feasibility of treating aluminum honeycomb sandwich panels as equivalent homogeneous panels for the electromagnetic force calculation, an experimental setup is established, as shown in Figure 8. A control motor drives an aluminum honeycomb sandwich panel to rotate at a certain speed. The parameters of the tested aluminum honeycomb sandwich panel are shown in Table 3. A cylindrical permanent magnet is located above the tested aluminum honeycomb sandwich panel, and its position can be adjusted by the horizontal and vertical linear slides. The height and diameter of the cylindrical permanent magnet are 30 mm, and its residual magnetism is about 1.42 T. A digital force gauge measures the magnitude of the electromagnetic force on the permanent magnet in the direction of relative motion velocity. According to Newton’s third law, the force measured is equal to the electromagnetic force on the aluminum honeycomb sandwich panel.

### 5.2. Experiment Results

According to Equation (20), the equivalent conductivity tensor of the tested aluminum honeycomb sandwich panel can be calculated as

(25) $$\bar{\sigma }\;=\;\left[\begin{array}{ccc} 6.55 & 0 & 0\\ 0 & 6.88 & 0\\ 0 & 0 & 7.65 \end{array}\right]\times 10^{5}\;S/m$$

**Figure 9:**
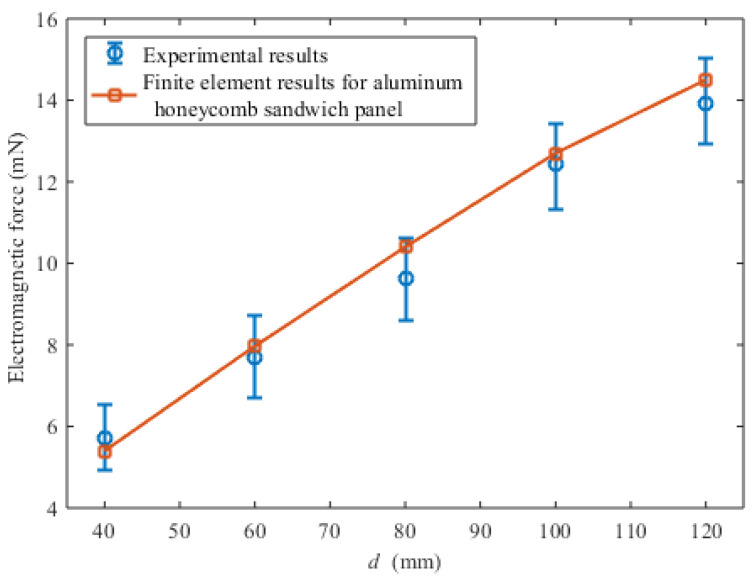
Experimental and finite element results of the electromagnetic force.

The height of the magnet from the honeycomb panel is 40 mm. The control motor drives the tested aluminum honeycomb sandwich panel rotating at a constant speed of 250 n/s. The experimental and finite element results of the electromagnetic force are shown in Figure 9. It can be seen that the finite element results using the equivalent conductivity tensor are consistent with the experimental results.

## 6. Conclusions

This paper provides an equivalent conductivity model for aluminum honeycomb sandwich panels. The calculation of the electromagnetic force acting on the aluminum honeycomb sandwich panel moving in a magnetic field can be simplified by treating the aluminum honeycomb sandwich panel as a homogeneous panel based on the proposed equivalent conductivity model. The numerical simulation results show that the homogeneous equivalent model considering an aluminum honeycomb core can improve the accuracy of induced electromagnetic forces compared to the previous methods that only consider the skins, especially when the skins are made of CFRP. In addition, the feasibility of treating aluminum honeycomb sandwich panels as homogeneous panels for calculating induced electromagnetic forces has been experimentally proven.

## Figures and Tables

**Table 2:** Parameter values in the numerical models.

Parameter	Value
Length of aluminum honeycomb sandwich panel, *L*	100 mm
Width of aluminum honeycomb sandwich panel, *B*	100 mm
Height of aluminum honeycomb sandwich panel, *H*	20 mm
Skin thickness, $h_{1}$	0.6 mm
Foil thickness, τ	0.06 mm
Cell length, *l*	3 mm
Diameter of permanent magnet, $a_{m}$	20 mm
Height of permanent magnet, $h_{m}$	20 mm
Remanence of magnet, $B_{r}$	1.45 T
Relative moving speed, *v*	1 m/s
Relative permeability of aluminum and air	1

**Table 3:** Parameter values in the experiments.

Parameter	Value
Length of aluminum honeycomb sandwich panel, *L*	400 mm
Width of aluminum honeycomb sandwich panel, *B*	400 mm
Height of aluminum honeycomb sandwich panel, *H*	21 mm
Skin thickness, $h_{1}$	0.3 mm
Foil thickness, τ	0.03 mm
Cell length, *l*	5 mm
Conductivity of the skins (LY12)	1.9 × 10${}^{7}$ S/m
Conductivity of the foil (LF2Y)	2.06 × 10${}^{7}$ S/m

## Data Availability

Not applicable.

## Abbreviations

The following abbreviations are used in this manuscript:

CFRP Carbon Fiber-Reinforced Plastic
